# The Simultaneous Detection of Dopamine and Uric Acid In Vivo Based on a 3D Reduced Graphene Oxide–MXene Composite Electrode

**DOI:** 10.3390/molecules29091936

**Published:** 2024-04-24

**Authors:** Lingjun Shang, Ruijiao Li, Haojie Li, Shuaiqun Yu, Xuming Sun, Yi Yu, Qiongqiong Ren

**Affiliations:** School of Medical Engineering, Xinxiang Medical University, Xinxiang 453003, China; slj1216002129@163.com (L.S.); li_4877@163.com (R.L.); 18436264383@163.com (H.L.); m15225998391@163.com (S.Y.); sunxuming@xxmu.edu.cn (X.S.); yuyi@xxmu.edu.cn (Y.Y.)

**Keywords:** dopamine, uric acid, reduced graphene oxide, MXene, simultaneous detection, in vivo

## Abstract

Dopamine (DA) and uric acid (UA) are essential for many physiological processes in the human body. Abnormal levels of DA and UA can lead to multiple diseases, such as Parkinson’s disease and gout. In this work, a three-dimensional reduced graphene oxide–MXene (3D rGO-Ti_3_C_2_) composite electrode was prepared using a simple one-step hydrothermal reduction process, which could separate the oxidation potentials of DA and UA, enabling the simultaneous detection of DA and UA. The 3D rGO-Ti_3_C_2_ electrode exhibited excellent electrocatalytic activity towards both DA and UA. In 0.01 M PBS solution, the linear range of DA was 0.5–500 µM with a sensitivity of 0.74 µA·µM^−1^·cm^−2^ and a detection limit of 0.056 µM (S/N = 3), while the linear range of UA was 0.5–60 µM and 80–450 µM, with sensitivity of 2.96 and 0.81 µA·µM^−1^·cm^−2^, respectively, and a detection limit of 0.086 µM (S/N = 3). In 10% fetal bovine serum (FBS) solution, the linear range of DA was 0.5–500 µM with a sensitivity of 0.41 µA·µM^−1^·cm^−2^ and a detection limit of 0.091 µM (S/N = 3). The linear range of UA was 2–500 µM with a sensitivity of 0.11 µA·µM^−1^·cm^−2^ and a detection limit of 0.6 µM (S/N = 3). The modified electrode exhibited advantages such as high sensitivity, a strong anti-interference capability, and good repeatability. Furthermore, the modified electrode was successfully used for DA measurement in vivo. This could present a simple reliable route for neurotransmitter detection in neuroscience.

## 1. Introduction

Dopamine (DA) and uric acid (UA) often coexist in human physical fluids and play key roles in physiological performance. As a neurotransmitter, DA plays a critical role in learning, attention, and memory [1]. Abnormal levels of DA affect the functions of the kidneys, cardiovascular system, and nervous system; metabolism; and motor function [2]. Its concentration is used as a parameter in the diagnosis of Parkinson’s disease, schizophrenia, and other neurogenic disorders [2,3,4,5]. UA is the final product of purine metabolism in the urine and blood, and its levels are responsible for gout, hyperuricemia, Lesch–Nyhan syndrome, and renal disease [6]. The concentrations of UA and DA in serum are 120–450 µM and 0.01–1 µM, respectively [7]. The level of UA is significantly correlated with the severity of DA damage in the striatum and the uptake of dopamine transporter in the substantia nigra, playing a neuroprotective role [8,9]. Furthermore, there is a significant correlation between DA and UA during the circadian cycle [10]. Hence, it is crucial to accurately detect DA and UA in clinical analysis and physiological research.

DA and UA exhibit high electrochemical activity, and electrochemical methods for their detection have the advantages of convenience, speed, low cost, and sensitivity [11]. During electrocatalysis, the accumulation of oxidation production at the surface can cause fouling and reduce sensitivity [12]. The development of electrode modification materials with excellent performance has attracted widespread attention in the field of electrochemical sensors.

When DA and UA come into contact with active nanomaterials, they can be detected simultaneously with great sensitivity, despite their very close oxidation potentials [13]. Due to their high electrocatalytic activity for redox reactions, carbon-based nanomaterials (such as graphene) have been widely used for the simultaneous determination of DA and UA [14]. Graphene is an emerging two-dimensional (2D) honeycomb lattice carbon-based nanomaterial with excellent electrical conductivity, great chemical durability, a large specific surface area, superior thermal stability, a wide electrochemical window, and good biocompatibility [15,16,17,18,19]. The π-π stacking interactions between individual graphene sheets may lead to irreversible aggregation [20]. This can result in a decrease in the performance of graphene. Assembling 2D flakes into a 3D architecture can effectively address this issue. Yang et al. used ERGO/GCE successfully to monitor DA, AA, and UA in urine samples [11]. Jiang et al. used 3D-doped graphene networks successfully to detect UA, AA, and DA in urine samples [15].

The introduction of 2D active nanosheets into 3D structures through different interfacial interactions, such as hydrogen bonding, π-π bridging, ionic bonding, and covalent bonding, can improve the biosensor detection performance [21,22]. MXene is a 2D titanium carbide with negatively charged function groups (-F, -OH, and -O) on its surface, and it exhibits excellent electrical conductivity, good stability, and hydrophilicity [23,24]. MXene has potential applications in electrochemical sensing. Zheng et al. used MXene successfully to detect DA in human serum samples with high selectivity against AA, UA, and glucose [25]. Xue et al. used Ti_3_C_2_T_X_/PtNPs successfully to detect DA coexisting with AA and UA [23]. However, MXene exhibits the issue of re-stacking, which hinders electrolyte penetration and decreases its electrochemical performance [22]. Introducing 2D MXene into a 3D rGO network structure can effectively address this issue [26]. This introduction prevents re-stacking and enlarges the specific surface area. Wang et al. used 3D porous laser-scribed graphene (LSG)–MXene to enhance the catalytic performance [14]. The electrode exhibited low detection limits of 0.13 µM DA and 0.47 µM UA.

In this work, we developed a three-dimensional reduced graphene oxide–MXene (3D rGO-Ti_3_C_2_) electrode, which could be used for the simultaneous detection of DA and UA. The electrode exhibited excellent anti-interference, stability, reproducibility, and good biocompatibility. Moreover, the 3D rGO-Ti_3_C_2_ electrode could be applied for the detection of UA and DA in serum samples. Finally, in vivo detection of DA in rat brains was achieved.

## 2. Results and Discussion

### 2.1. Characterization

**Figure 1:**
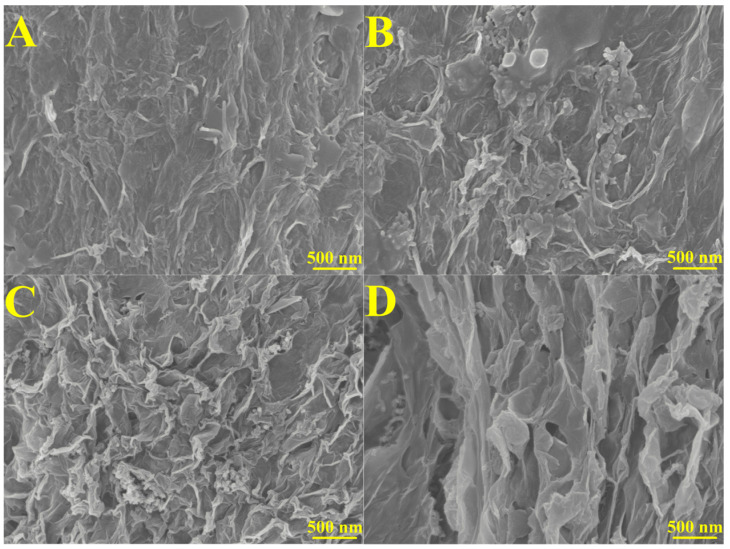
SEM images of (**A**) 3D rGO, (**B**) 3D rGO-Ti_3_C_2_ (3:1), (**C**) 3D rGO-Ti_3_C_2_ (2:1), and (**D**) 3D rGO-Ti_3_C_2_ (1:1).

SEM images were taken to investigate the surface of the 3D rGO and 3D rGO-Ti_3_C_2_ electrodes. The SEM images of the 3D rGO showed a three-dimensional porous structure and characteristic graphene folds, which increased the surface area, provided active sites for electron transfer, and allowed for free diffusion of the electrolytes within the framework (Figure 1A) [15]. Figure 1B–D show the SEM images of the 3D rGO-Ti_3_C_2_ membranes at different ratios (3:1, 2:1, and 1:1). As the Ti_3_C_2_ content increases, the folds of graphene decrease, and the membrane becomes denser. In the case of the 1:1 ratio, more porous structures were observed. Hydrogen bonding and electrostatic interactions reduced the self-stacking of the graphene and Ti_3_C_2_, increased the interlayer spacing, enhanced the presence of active sites, shortened the ion diffusion path, and improved the catalytic activity [27]. Further, XRD and XPS characterizations are shown in Figure S1.

### 2.2. Direct Electrochemical Behavior of the 3D rGO-Ti_3_C_2_ Electrode

**Figure 2:**
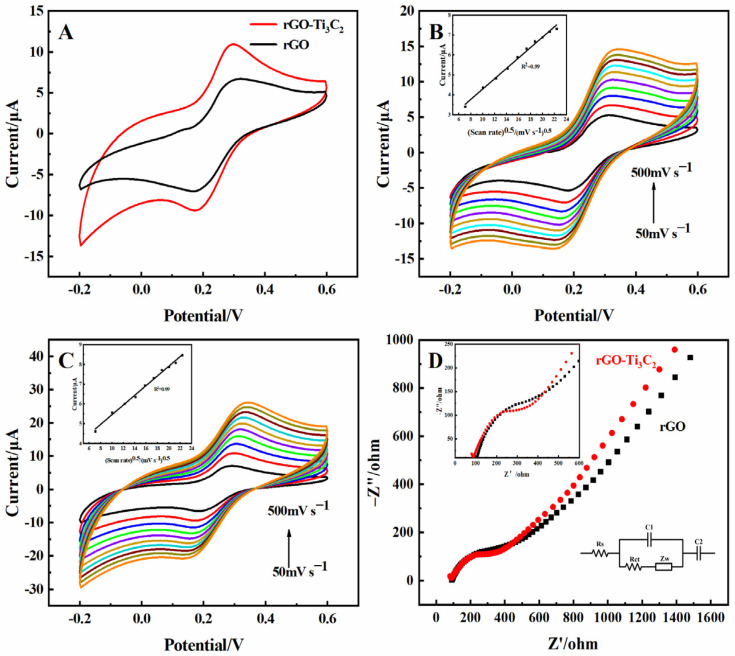
(**A**) CV of the 3D rGO and 3D rGO-Ti_3_C_2_ electrodes in 5 mM K_3_[Fe(CN)_6_] (0.1 M KCl). Scan rate: 100 mV·s^−1^. CV of the (**B**) 3D rGO and (**C**) 3D rGO-Ti_3_C_2_ electrodes in 5 mM K_3_[Fe(CN)_6_] (0.1 M KCl) at scan rates of 50, 100, 150, 200, 250, 300, 350, 400, 450, 500 mV·s^−1^ (inset: plot of the oxidation peak currents vs. square root of scan rates). (**D**) EIS diagrams of the rGO and 3D rGO-Ti_3_C_2_ electrodes in 5 mM K_3_[Fe(CN)_6_] (0.1 M KCl). Frequency range: 0.1–10^5^ Hz (inset: equivalent circuit and amplification diagram of high frequency).

Cyclic voltammetry (CV) was performed to characterize the electron process and effective surface area of the 3D rGO and 3D rGO-Ti_3_C_2_ electrodes. As shown in Figure 2A, a pair of obvious redox peaks was observed for the 3D rGO electrode. In comparison, the redox peak currents of the rGO-Ti_3_C_2_ electrode increased significantly. This can be attributed to the incorporation of Ti_3_C_2_, which enhances the effective surface area of the electrode and boosts the electron transfer rate. The oxidation peak currents showed a linear increase with the square root of the scan rate, as shown in Figure 2B,C. The correlation coefficient (R^2^) for both relationships was 0.99, indicating a typical diffusion-controlled quasi-reversible electron transfer process. The effective surface area of the 3D rGO and 3D rGO-Ti_3_C_2_ electrodes could be determined by applying the Randles–Sevcik equation:

I*_p_* = 2.69 × 10^5^ A*n*^3/2^ D_0_^1/2^ C_0_ *v*^1/2^

where I*_p_* represents the peak current, A represents the effective surface area, *n* indicates the number of electrons involved in the electrochemical process (*n* = 1), D_0_ represents the diffusion coefficient (D_0_ = 0.673 × 10^−5^ cm^2^·s^−1^), C_0_ represents the concentration (C_0_ = 5 × 10^−6^ mol·cm^−3^), and *v* denotes the scan rate. Using this equation, the effective surface area of the rGO and rGO-Ti_3_C_2_ electrodes was 0.023 cm^2^ and 0.027 cm^2^, respectively.

Electrochemical impedance spectroscopy (EIS) was performed to analyze the electron transfer properties of the 3D rGO and 3D rGO-Ti_3_C_2_ electrodes. In the EIS spectrum, the high-frequency intercept on the x-axis represents the solution resistance [18], the high-frequency semicircle represents the charge transfer resistance (Rct), and the linear portion at low frequencies represents the Warburg impedance (Zw). As shown in Figure 2D, the solution resistance is approximately 91 Ω, and the Rct values for the 3D rGO electrode and the 3D rGO-Ti_3_C_2_ electrode are 174.02 Ω and 114.6 Ω, respectively. This result indicated that Ti_3_C_2_ enhances the conductivity of the electrode and accelerates the electron transfer rate of the electrochemical reaction.

### 2.3. Electrocatalytic Oxidation of DA and UA by the 3D rGO-Ti_3_C_2_ Electrode

**Figure 3:**
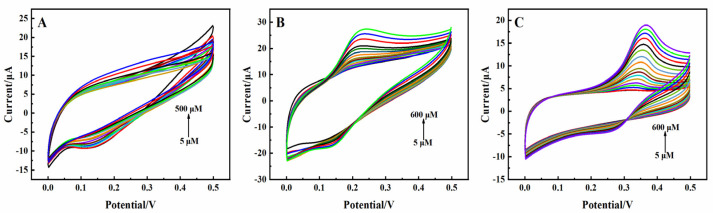
CV of the 3D rGO-Ti_3_C_2_ electrode at different concentrations of (**A**) AA, (**B**) DA, and (**C**) UA in 0.01 M PBS (pH = 7.0). Scan rate: 100 mV·s^−1^. Potential range: 0.0–0.5 V.

The electrocatalytic properties of the modified electrodes in terms of DA, UA, and AA oxidation were first investigated according to CV. The response currents to different concentrations of AA, DA, and UA are shown in Figure 3. The 3D rGO-Ti_3_C_2_ electrode exhibited a minimal response to AA because of the homogeneous negative charges of AA and Ti_3_C_2_, which repel each other [23,28]. DA and UA exhibited quasi-reversible electrochemical behavior, with distinct oxidation peaks observed at 0.22 V and 0.36 V, respectively. rGO has aromatic rings with richly delocalized π electrons which function as excellent electron collectors and transporters [29]. The oxygen-containing groups on rGO could form selective interfaces through hydrogen bonding with the protonated groups of DA and UA [29,30]. The rGO-Ti_3_C_2_ composite exhibited high catalytic activity via Ti-O-C covalent bonds and π-π bridging interactions [21]. This could be beneficial for catalyzing the oxidation of DA and UA. The oxidation pathways of DA and UA are expressed in Scheme S1. These results indicated that the 3D rGO-Ti_3_C_2_ electrode exhibited great selectivity and could detect DA and UA simultaneously.

**Figure 4:**
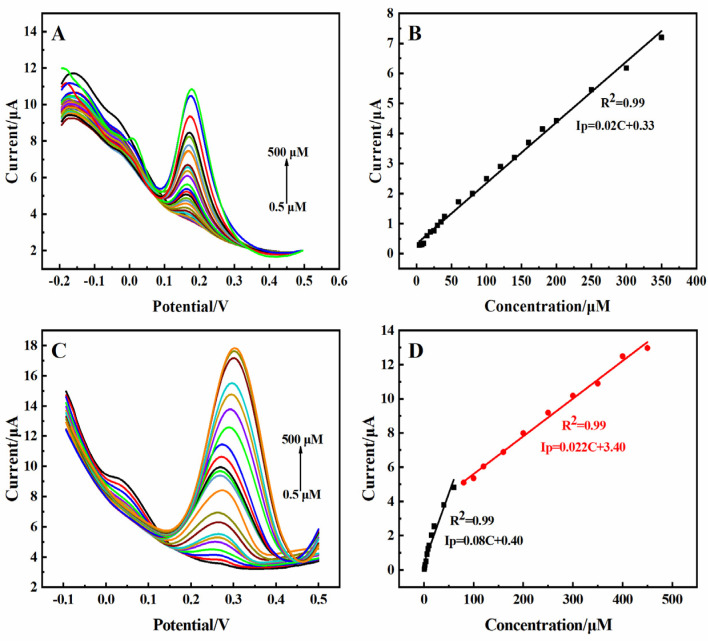
DPV of the 3D rGO-Ti_3_C_2_ electrode at different concentrations of (**A**) DA and (**C**) UA in 0.01 M PBS (pH = 7.0). Calibration curve of current response vs. (**B**) DA and (**D**) UA concentration.

The differential pulse voltammetry (DPV) response to the electrocatalytic oxidation of DA and UA showed obvious oxidation peaks at 0.175 V and 0.3 V, respectively (Figure 4A,C). The current response showed excellent linear correlation in the concentration range of 0.5–500 µM to DA (Figure 4B) and in the concentration ranges of 0.5–60 µM and 80–450 µM to UA (Figure 4D), respectively. The sensitivity of DA was 0.74 µA·µM^−1^·cm^−2^, and the sensitivity of UA was 2.96 µA·µM^−1^·cm^−2^ and 0.81 µA·µM^−1^·cm^−2^, respectively. The detection limits for DA and UA were 0.061 µM and 0.086 µM (S/N = 3), respectively. The amperometric responses of the 3D rGO-Ti_3_C_2_ electrode to DA and UA were also recorded, as shown in Figure S4. The calculated data for both the DPV and amperometric responses showed almost identical results, which demonstrated the excellent repeatability and reliability of the constructed 3D rGO-Ti_3_C_2_ composite electrode.

**Figure 5:**
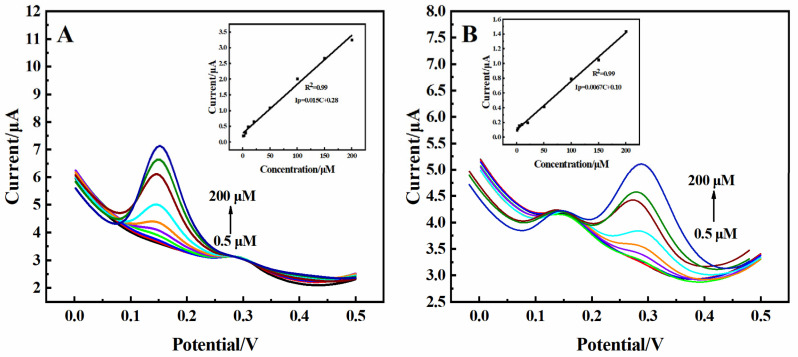
(**A**) DPV of the 3D rGO-Ti_3_C_2_ electrode at different concentrations of DA with 20 µM UA in 0.01 M PBS (inset: calibration curve of current response vs. DA concentration). (**B**) DPV of the 3D rGO-Ti_3_C_2_ electrode at different concentrations of UA with 20 µM DA in 0.01 M PBS (inset: calibration curve of current response vs. UA concentration).

DA and UA were simultaneously determined according to DPV in 0.01 M PBS solution. As shown in Figure 5A, the peak current of DA increased with an increasing concentration of DA when keeping the concentration of UA constant (20 µM). The addition of DA had almost no effect on the peak potential of UA. The current response showed a linear relationship with the concentration of DA in the range of 0.5–200 µM (Figure 5A inset). The sensitivity was determined to be 0.59 µA·µM^−1^·cm^−2^, and the determination was 0.065 µM (S/N =3). As shown in Figure 5B, the peak current of UA increased with an increasing concentration of UA in a solution with DA (20 µM). The continuous addition of UA had almost no effect on the peak potential of DA. It was found that the response current had a good linear relationship with the concentration of UA in the range of 0.5–200 µM, with a sensitivity of 0.25 µA·µM^−1^·cm^−2^ and a detection limit of 0.1 µM (S/N = 3) (Figure 5B inset). These results indicated that 3D rGO-Ti_3_C_2_ composite electrodes are suitable for the simultaneous detection of DA and UA.

### 2.4. Anti-Interference, Stability, and Repeatability of the 3D rGO-Ti_3_C_2_ Electrode

**Figure 6:**
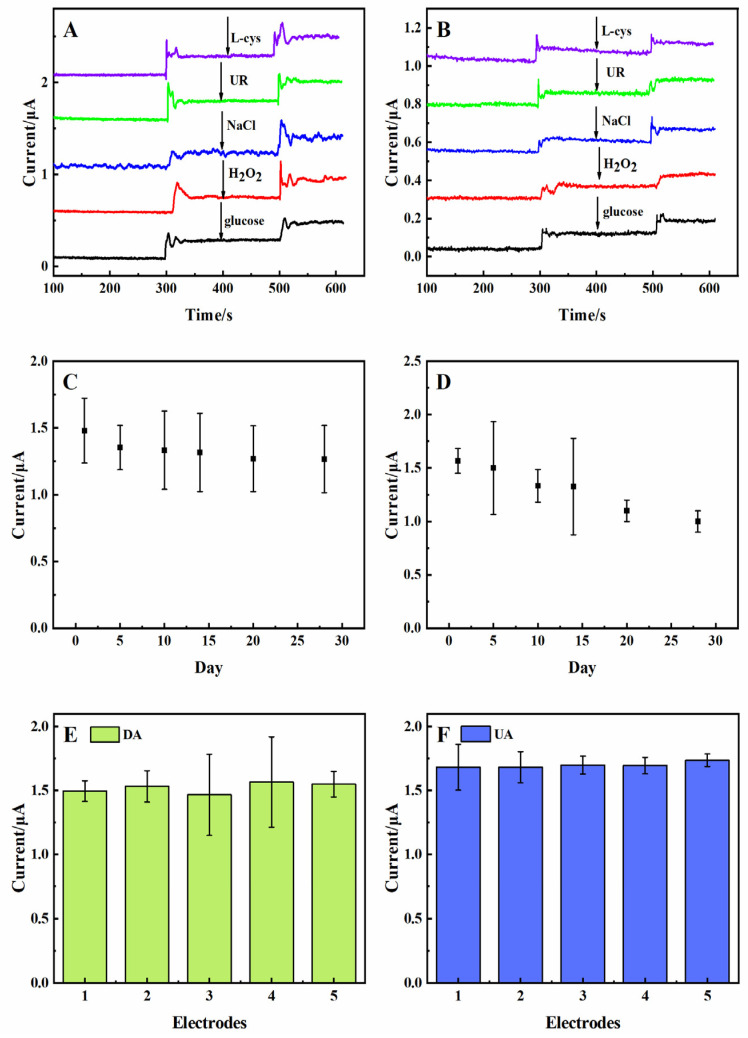
Anti-interference of the rGO-Ti_3_C_2_ electrode with (**A**) DA and (**B**) UA in 0.01 M PBS at constant potentials of 0.175 V and 0.3 V, respectively. Long-term (30 d) stability of the 3D rGO-Ti_3_C_2_ electrodes with 50 µM of (**C**) DA and (**D**) UA in 0.01 M PBS. Repeatability study of five 3D rGO-Ti_3_C_2_ electrodes with 50 µM of (**E**) DA and (**F**) UA.

Inorganic/organic compounds and AA often coexist with DA and UA in tissue fluids, serum, and the central nervous system [14]. It was necessary to analyze the anti-interference of the 3D rGO-Ti_3_C_2_ electrode. As shown in Figure 6A, the response current was recorded upon the successive addition of 10 µM of DA, 10 µM of interferents (L-cys, UR, NaCl, H_2_O_2_, and glucose), and then 10 µM of DA again in 0.01 M PBS solution at a constant potential of 0.175 V. The modified electrode had no response to the interferents, while a significant response was observed to the subsequent addition of DA. The same work proceeded in terms of the response to UA (Figure 6B). These results suggested that the presence of interferents would not interfere with the detection of DA and UA. The 3D rGO-Ti_3_C_2_ electrode had a good anti-interference ability.

Moreover, regular monitoring of the DPV response to 50 µM of DA and UA was conducted (Figure 6C,D). After 30 d, the oxidation currents of DA and UA were maintained at 86% and 70%, respectively. The results confirmed the great long-term stability of the 3D rGO-Ti_3_C_2_ electrode. Measurement of the repeatability of the 3D rGO-Ti_3_C_2_ electrode was investigated using a DPV study with five 3D rGO-Ti_3_C_2_ electrodes prepared using the same method and tested with 50 µM of DA and UA (Figure 6E,F). The current responses were very similar for all the electrodes. The relative standard deviation (RSD) of the response of the electrodes to DA and UA was 0.027 and 0.013, respectively. These analyses indicated that the 3D rGO-Ti_3_C_2_ electrode exhibited excellent anti-interference, long-term stability, and repeatability.

### 2.5. Fetal Bovine Serum

**Figure 7:**
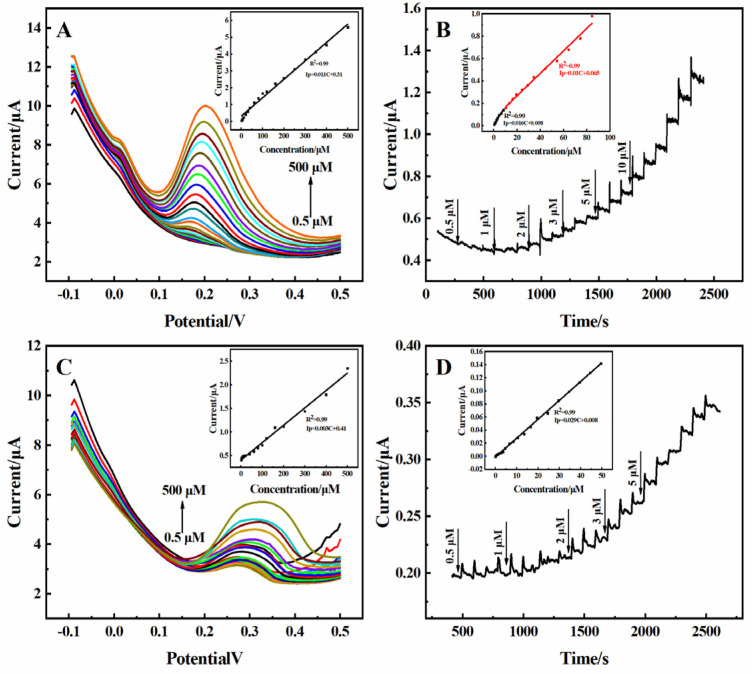
The (**A**) DPV and (**B**) amperometric response of the 3D rGO-Ti_3_C_2_ electrodes in 10% FBS solution at different concentrations of DA (inset: calibration curve of current response vs. DA concentration). The (**C**) DPV and (**D**) amperometric response of the 3D rGO-Ti_3_C_2_ electrodes in 10% FBS solution at different concentrations of UA (inset: calibration curve of current response vs. UA concentration).

The reliability of the practical application of the 3D rGO-Ti_3_C_2_ electrode was studied. The 3D rGO-Ti_3_C_2_ electrode was subjected to DPV, CV, and amperometric response analysis with DA and UA in 10% fetal bovine serum (FBS) solution (prepared in 0.01 M PBS). As shown in Figure 7A, the current response increased with an increasing concentration of DA. The calibration curve in the inset in Figure 7A demonstrates the linear relationship between the current response and the concentration of DA in the range of 0.5–500 µM, with a sensitivity of 0.41 µA·µM^−1^·cm^−2^. The determination was 0.091 µM. As shown in Figure 7B, the current response exhibited a good linear relationship with the concentration of DA, with linear ranges of 0.5–8 µM and 10–80 µM. The sensitivities were 0.61 µA·µM^−1^·cm^−2^ and 0.41 µA·µM^−1^·cm^−2^, and the determination was 0.087 µM. The sensitivity of the 3D rGO-Ti_3_C_2_ electrode to DA in 10% FBS solution was similar in terms of both the DPV and amperometric responses. However, compared to the sensitivity in the 0.01 M PBS (0.74 µA·µM^−1^·cm^−2^) solution, there was a decrease in sensitivity in the 10% FBS solution. This may because the oxidation peak potential of DA in the 10% FBS solution (0.3 V) shifted to a higher value compared to that in the 0.01 M PBS solution (0.22 V) (Figure S5). Identical experiments were carried out in terms of the response to UA in 10% FBS solution, and similar results were obtained (Figure 7C,D). A comparison of the 3D rGO-Ti_3_C_2_ electrode’s sensitivity is shown in Table 1. Although there is a slight decrease in sensitivity, these results indicate that the developed 3D rGO-Ti_3_C_2_ composite electrode can be used for the detection of DA and UA in serum. The results in Table 2 show that the 3D rGO-Ti_3_C_2_ electrode has a wider linear range and a lower detection limit, which is more suitable for the simultaneous determination of DA and UA in a variety of environments.

### 2.6. Detection of DA in Rat Brains

Due to the complexity of the brain, cells or proteins may be non-specifically adsorbed onto the surface of the 3D rGO-Ti_3_C_2_ electrode after its implantation, resulting in a decrease in its sensing performance [35]. Therefore, bovine serum albumin (BSA) was used to mimic protein adsorption to detect the anti-biofouling properties of the 3D rGO-Ti_3_C_2_ electrode in vivo. The 3D rGO-Ti_3_C_2_ electrode was immersed in 40 mg·mL^−1^ of BSA (0.01 M PBS) solution for 2 h. The sensitivity of the electrode was maintained at 73% of its initial value (Figure S6). This maintenance of its sensitivity was because of the hydrophilic surface of Ti_3_C_2_, which decreased the protein adsorption [2]. This result indicated that the complex environment in the brain has a minimal impact on the performance of the modified electrode.

**Figure 8:**
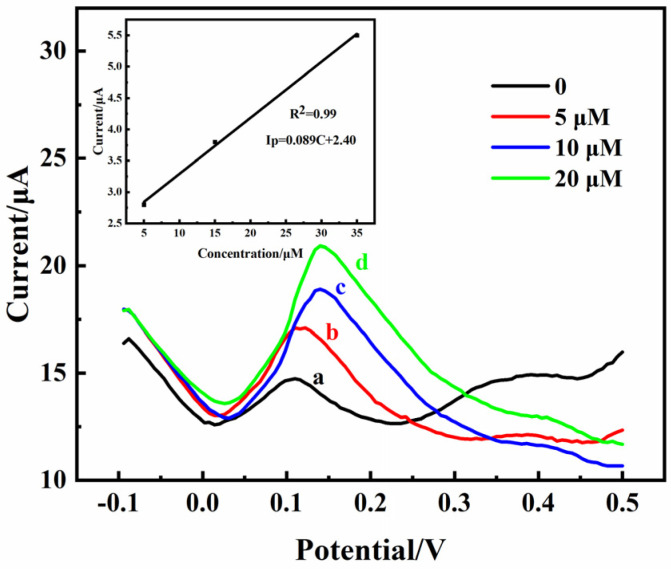
DPV of the 3D rGO-Ti_3_C_2_ electrode at different concentrations (a: 0 µM, b: 5 µM, c: 10 µM and d: 20 µM) of DA in the rat striatum (inset: calibration curve of current response vs. DA concentration).

An abnormal DA content in the striatum can contribute to the development of Parkinson’s disease [2]. Therefore, monitoring striatum DA levels in vivo is crucial. The practicality of the 3D rGO-Ti_3_C_2_ electrode was studied according to intracerebral microperfusion. A characteristic peak (2.5 µA) of DA appeared at 0.175 V during the DPV scanning without the addition of DA because DA exists in the striatum itself (Figure 8a). This result indicated that the 3D rGO-Ti_3_C_2_ electrode was correctly implanted into the striatum of the rat brains. Different concentrations (5, 10, and 20 µM) of DA were injected into the rat striatum at a slow rate, and the DPV signals were subsequently recorded. As the concentration of DA increased, the current response gradually increased. According to the calibration curve equation in Figure 8’s inset, the actual DA concentration in the rat striatum without a DA injection was 1.1 µM. The rats’ scalps were then sutured. After 4 weeks, the rats were in good health, which indicated that the 3D rGO-Ti_3_C_2_ electrode has good biocompatibility, is non-toxic, and is suitable for monitoring DA in vivo.

## 3. Materials and Methods

### 3.1. Chemicals and Materials

The dopamine (DA, 98%), uric acid (UA, 99%), ascorbic acid (AA, 99%), D-glucose monohydrate (99.5%), NaCl (99.9%), H_2_O_2_ (30%), KCl (99.5%), potassium ferricyanide (K_3_[Fe(CN)_6_], 99.5%), and L-cysteine (99%) were purchased from Macklin (Shanghai, China). All the reagents were analytically pure. The graphene oxide (GO) was bought from Ashine Advanced Carbon Materials Co., Ltd. (Changzhou, China). The MXene (Ti_3_C_2_) was obtained from Beike 2D Materials Co., Ltd. (Beijing, China). The Apiezon Wax W was bought from Madison Technology Co., Ltd. (Beijing, China). The isoflurane and brain stereotaxic instruments were bought from PWD Life Science Co., Ltd. (Shenzhen, China).

### 3.2. Preparation of the 3D rGO-Ti_3_C_2_ Electrode

The 3D rGO-Ti_3_C_2_ electrodes were prepared using a one-step hydrothermal reduction method. A mixture of GO (0.3 mg·mL^−1^) and Ti_3_C_2_ (0.3 mg·mL^−1^) at ratios of 1:1, 2:1, and 3:1 (*v*/*v*) was ultrasonically dispersed for 1 h. Then, a copper wire (diameter of 0.1 mm) and the resulting suspension were sealed in a 5 mL Teflon-lined autoclave and maintained at 180 °C for 3 h. The obtained rGO-Ti_3_C_2_ hydrogel was dried in the air. Finally, the sides of the rGO-Ti_3_C_2_ were encapsulated in wax. The 3D rGO electrode was prepared using the same method.

### 3.3. Electrochemical Measurements

All the electrochemical measurements were conducted using a three-electrode system (CHI660e, Shanghai, China), which included the rGO-Ti_3_C_2_ electrode, Ag/AgCl, and Pt wire, as the working, reference, and counter electrodes, respectively. The morphology of the modified electrode was investigated using scanning electron microscopy (SEM, Nova 450; FEI Inc., Eindhoven, The Netherlands). X-ray diffraction (XRD, SmartLab, Akishima, Japan) was used to analyze the structure of the modified electrodes. X-ray photoelectron spectroscopy (XPS, ESCALAB Xi+, Thermo Fisher Scientific, Waltham, MA, USA) was used to characterize the elemental composition of the 3D rGO-Ti_3_C_2_ electrode.

### 3.4. Animals

All the rats (SD male, 7–8 weeks old) were purchased from Henan SCBS Biotechnology Co., Ltd. The experimental protocols received approval from the Animal Experimentation Committee of Xinxiang Medical University. As shown in Figure S7, during the test, the rats went under deep anesthesia. The rats were then secured using a brain stereotaxic instrument, and their scalps were removed. The 3D rGO-Ti_3_C_2_ electrode was implanted into the striatum (AP = 0.0 mm, L = 2.0 mm from bregma, V = 2.5 mm from dura). Pt wire was implanted into nearby small holes. The Ag/AgCl was implanted into the opposite striatum. All three electrodes were fixed using dental silicate cement. A hole was opened 2 mm away from the 3D rGO-Ti_3_C_2_, and a micro-sample syringe was inserted.

## 4. Conclusions

A 3D rGO-Ti_3_C_2_ composite electrode was successfully fabricated and employed for the electrochemical simultaneous detection of DA and UA. The modified electrode was successfully used for electrochemical monitoring of DA in rat striatum. The electrochemical data suggested that Ti_3_C_2_ enhances the conductivity of the electrode and accelerates the electron transfer rate of the electrochemical reaction. The 3D rGO-Ti_3_C_2_ composite electrode exhibited enhanced electrocatalytic activities and demonstrated high sensitivity, excellent selectivity, remarkable stability, and satisfactory repeatability. The modified electrode was found to be well suited to the detection of DA and UA in serum. After the implantation of the 3D rGO-Ti_3_C_2_ composite electrode into the rat striatum, it exhibited great biocompatibility and showed great responses to DA. This work may present a simple, reliable route for monitoring in neuroscience.

## Figures and Tables

**Table 1:** Three-dimensional rGO-Ti_3_C_2_ electrode sensitivity comparison.

Samples	Electrochemical Method	Detection Object	Linear Range (µM)	Detection Limit (µM)	Sensitivity (µA·µM^−1^·cm^−2^)
0.01 M PBS	DPV	DA	0.5–500	0.061	0.74
		UA	0.5–60, 80–450	0.086	2.96, 0.81
	Amperometric	DA	0.5–260	0.065	0.74
		UA	0.5–105	0.088	0.7
10% FBS	DPV	DA	0.5–500	0.091	0.41
		UA	2–500	0.6	0.11
	Amperometric	DA	0.5–8, 10–80	0.087	0.61, 0.41
		UA	0.5–50	0.25	0.107

**Table 2:** Simultaneous detection of DA and UA using different modified electrodes.

Electrode Materials	Linear Range (µM)		LOD (µM)		References
	DA	UA	DA	UA	
3DGH-Fc/GCE	10–180	8–400	0.042	0.067	[1]
MgO/Gr/Ta	0.1–7	1–70	0.15	0.12	[7]
Au-Pd/MXene/LSG	12–240	8–100	0.13	1.47	[14]
CNNS-GO	1–20	10–100	0.096	0.228	[29]
fg-C3N4/MWNTs/GO	2–100	4–200	0.22	1.36	[31]
CTAB-GO/MWNT/GCE	5–300	3–60	1.5	1	[32]
HNGA/GCE	0.6–75	0.4–50	0.22	0.12	[33]
RGO-ZnO/GCE	1–70	3–330	0.33	1.08	[34]
3D rGO-Ti_3_C_2_	0.5–500	0.5–450	0.061	0.085	This work

## Data Availability

The data are contained within the article.

## Supplementary Materials

The following supporting information can be downloaded at https://www.mdpi.com/article/10.3390/molecules29091936/s1. Figure S1: (A) XPS pattern of 3D rGO and 3D rGO-Ti_3_C_2_ (1:1). (B) XRD spectra of 3D; Figure S2: Effect of 3D rGO-Ti_3_C_2_ electrode at different pH on catalytic current and oxidation peak potential of 100 µM (A) DA and (B) UA in 0.01 M PBS solution; Figure S3: Current response values at the concentration of 50 µM of (A) DA and (B) UA for different ratios of rGO and Ti_3_C_2_ in 0.01 M PBS (pH = 7); Figure S4: (A) Amperometric response of the 3D rGO-Ti_3_C_2_ electrodes upon adding DA in 0.01 M PBS at a constant potential of 0.175 V under continuous stirring (inset: calibration curve of current response vs. DA concentration). (B) Amperometric response of the 3D rGO-Ti_3_C_2_ electrodes upon adding UA in 0.01 M PBS at a constant potential of 0.3 V under continuous stirring (inset: calibration curve of current response vs. UA concentration); Figure S5: CV of the 3D rGO-Ti_3_C_2_ electrodes at different concentrations of (A) DA and (B) UA in 10% FBS. Scan rate: mV·s^−1^. Potential range: 0.0–0.5 V; Figure S6: Current response of the 3D rGO-Ti_3_C_2_ electrodes before and after immersing in 40 mg·mL^−1^ BSA for DA; Figure S7: 3D rGO-Ti_3_C_2_ electrode, Pt wire, Ag/AgCl, and mico-sample syringe were implanted into rat striatum. Scheme S1: Oxidation pathways of DA and UA. Refs. [36,37] are cited in the Supplementary Materials.
