# Supplementary material for: The Simultaneous Detection of Dopamine and Uric Acid In Vivo Based on a 3D Reduced Graphene Oxide–MXene Composite Electrode
Source: Molecules. 2024 Apr 24;29(9):1936. doi: 10.3390/molecules29091936 (PMC11085402; doi:10.3390/molecules29091936)
Supplement: Supplementary file 1 [file molecules-29-01936-s001.zip › molecules-2959880-supplementary.pdf]

The Simultaneous Detection of Dopamine and Uric Acid In Vivo Based on a 3D Reduced Graphene Oxide–MXene Composite Electrode

Lingjun Shang, Ruijiao Li, Haojie Li, Shuaiqun Yu, Xuming Sun, Yi Yu and Qiongqiong Ren *

School of Medical Engineering, Xinxiang Medical University, Xinxiang 453003, China; slj1216002129@163.com (L.S.); li_4877@163.com (R.L.); 18436264383@163.com (H.L.); m15225998391@163.com (S.Y.); sunxuming@xxmu.edu.cn (X.S.); yuyi@xxmu.edu.cn (Y.Y.)

* Correspondence: 151036@xxmu.edu.cn; Tel.: +86-373-3831929

1. XRD and XPS characterization

As shown in Figure S1A, XPS measurements examined the elemental composition of 3D rGO films and 3D rGO-Ti_3C_2 (1:1) films. The results indicated that 3D rGO-Ti_3C_2 (1:1) contains C, O, Ti, and F elements, while rGO contains C and O. Figure S1B illustrated the structural analysis through XRD of rGO and 3D rGO-Ti_3C_2 (1:1), where the diffraction peaks near 2θ = 25.7° and 2θ = 43.5° correspond to the (002) and (100) planes of graphene-like structures, respectively [1]. Ti_3C_2 couldn't be modified on the electrode. Therefore, XRD of Ti_3C_2 was not carried out. The peak of $Ti_3C_2T_x$ MXene (2θ ~ 9°) disappears with the addition of rGO. Similar results have been reported by Zhang *et al.* [2]. Sun *et al.* increased the content of $Ti_3C_2T_x$ ($Ti_3C_2T_x$:rGO = 7:1), more complete peaks can display [3]. In our works, the mixing ration of $Ti_3C_2T_x$ and rGO is 1:1. Therefore, only a portion of the peaks are displayed.

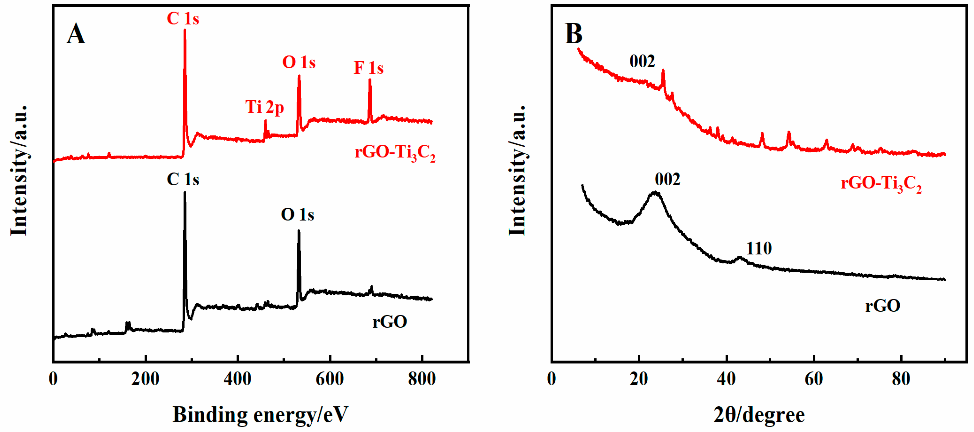

Figure S1. (A) XPS pattern of 3D rGO and 3D rGO-Ti_3C_2 (1:1). (B) XRD spectra of 3D rGO and 3D rGO-Ti_3C_2 (1:1).

2. Optimization of experimental conditions

To achieve optimal performance of the electrochemical sensor, pH and the ratio of 3D rGO-Ti_3C_2 composite material need to be optimized.

2.1. Influence of pH

As shown in Figure S2, the influence of the 3D rGO-Ti_3C_2 electrode on the electrocatalytic oxidation current and oxidation peak potential of 100 µM DA and UA within the pH range of 5.0−9.0 was evaluated using differential pulse voltammetry (DPV). With increasing pH, the oxidation peak potentials of DA and UA continuously decreased, indicating a deprotonation process occurring

during the oxidation process, which can enhance the interference resistance of the modified electrode. The current responses of DA and UA initially increased with increasing pH, reaching a peak value at pH 7.0. However, the current response decreased as the pH increased beyond 7.0. Therefore, a pH value of 7.0 in the 0.01 M PBS supporting electrolyte was chosen for the subsequent electrochemical analysis.

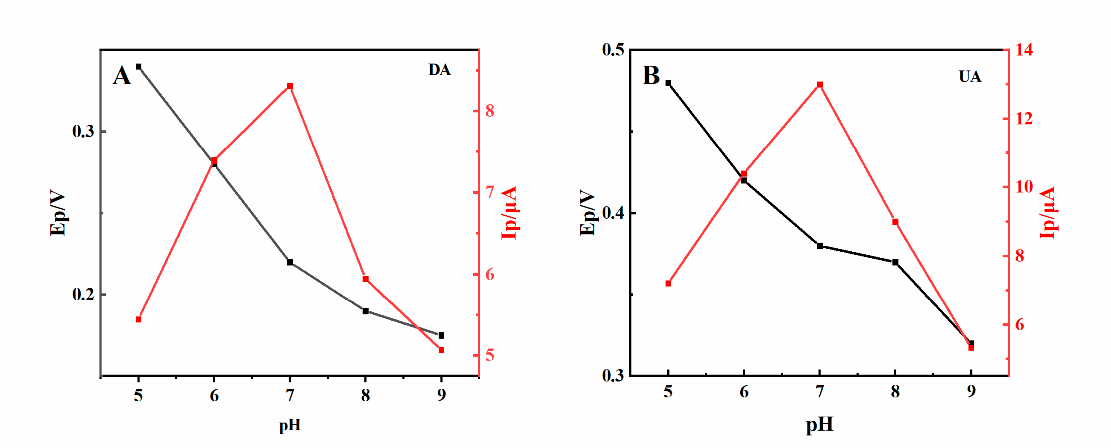

Figure S2. Effect of 3D rGO-Ti_3C_2 electrode at different pH on catalytic current and oxidation peak potential of 100 μM (A) DA and (B) UA in 0.01 M PBS solution.

2.2. Influence of rGO and Ti_3C_2 ratio

The electrocatalytic oxidation of 50 μM DA and UA was studied by DPV in 0.01 M PBS (pH = 7). In the DA solution, the DPV oxidation peak current increased with the increase of Ti_3C_2 content, reaching the maximum value when the ratio of rGO to Ti_3C_2 reached 1:1. Similarly, in the UA solution, an increase in Ti_3C_2 content increased the oxidation peak current. The currents reach maximum value at a ratio of rGO to Ti_3C_2 of 1:1 (Figure S3A and B). The continuous increase of Ti_3C_2 content prevented the formation of complete hydrogel on the copper wire surface. The improvement in electrode performance can be attributed to the synergistic effect between rGO and Ti_3C_2. The combination of rGO and Ti_3C_2 enhances conductivity, increases surface area, and enhances electrochemical performance. Therefore, the optimal ratio of rGO to Ti_3C_2 is 1:1. In this experiment, the copper wire only serves as a conductor.

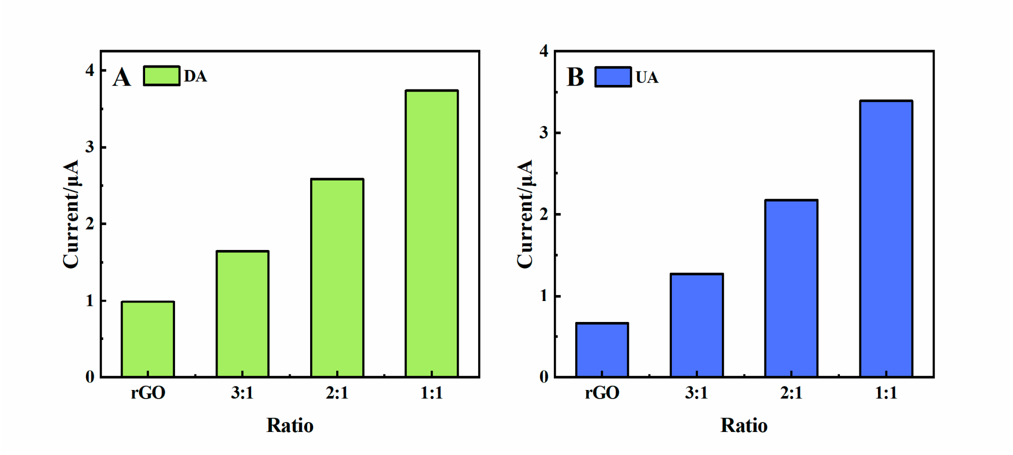

Figure S3. Current response values at the concentration of 50 μM of (A) DA and (B) UA for different ratios of rGO and Ti_3C_2 in 0.01 M PBS (pH = 7).

3. Oxidation pathways of DA and UA

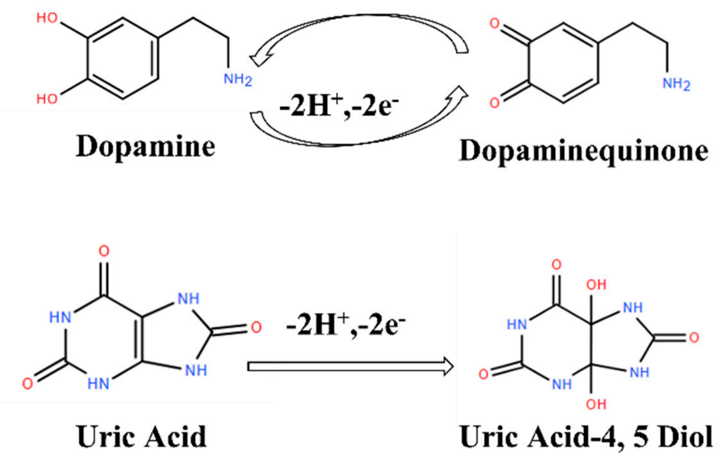

Scheme S1. Oxidation pathways of DA and UA.

4. Amperometric response

To further investigate the electrocatalytic capability and real-time detection of the 3D rGO-Ti_3C_2 electrode to DA and UA, amperometric response measurements were conducted in 0.01 M PBS solution using different concentrations of DA and UA.

Figure S4A showed the amperometric response of 3D rGO-Ti_3C_2 electrode to DA at a potential of 0.175 V. When DA was added to the 0.01 M PBS, the current responded well within 3 s. The current response of 3D rGO-Ti_3C_2 electrode showed excellent linear correlation with the concentration range of 0.5–260 μM of DA (Figure S4A inset). The sensitivity of the electrode was 0.74 μA μM^{-1} cm^{-2}, and the detection limit was 0.056 μM (S/N = 3).

Figure S4B showed the amperometric response of 3D rGO-Ti_3C_2 electrode to UA at a potential

of 0.3 V. After adding different concentrations of UA to the continuously stirred electrolyte, the oxidation current of the modified electrode showed a rapid step-like response within 3 s. The current response of 3D rGO-Ti_3C_2 electrode showed a good linear correlation with the concentration range of 0.5−105 μM of UA (Figure S4B inset). The sensitivity of the modified electrode was 0.70 μA μM^{-1} cm^{-2}, and the detection limit was 0.088 μM (S/N = 3).

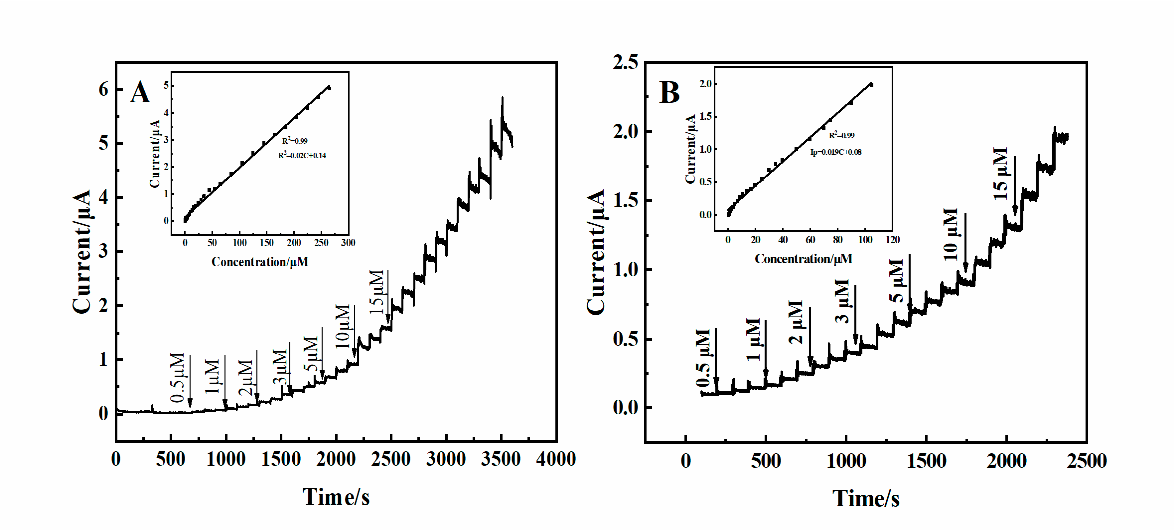

Figure S4. (A) Amperometric response of the 3D rGO-Ti_3C_2 electrodes upon adding DA in 0.01 M PBS at a constant potential of 0.175 V under continuous stirring (inset: calibration curve of current response vs. DA concentration). (B) Amperometric response of the 3D rGO-Ti_3C_2 electrodes upon adding UA in 0.01 M PBS at a constant potential of 0.3 V under continuous stirring (inset: calibration curve of current response vs. UA concentration).

5. CV analysis in 10% fetal bovine serum (FBS)

As shown in Figure S5, 3D rGO-Ti_3C_2 electrode was subjected by CV response analysis to DA and UA in 10% FBS solution.

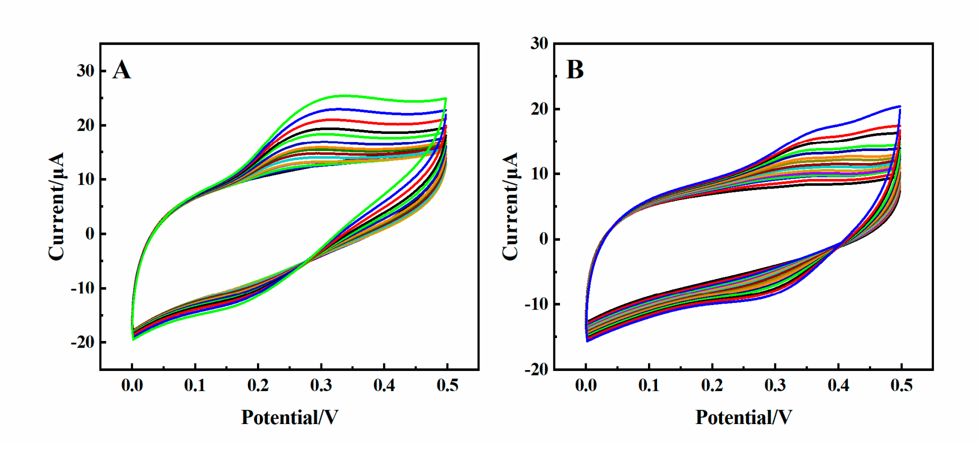

Figure S5. CV of the 3D rGO-Ti_3C_2 electrodes at different concentrations of (A) DA and (B) UA in

10% FBS. Scan rate: 100 mV s^{-1}. Potential range: 0.0−0.5 V.

6. anti-biofouling property

The current response of 3D rGO-Ti_3C_2 electrode immersed in a 40 mg mL^{-1} bovine serum albumin (BSA) solution (0.01 M PBS prepared) before and after 2 h was analyzed for DA as shown in Figure S6.

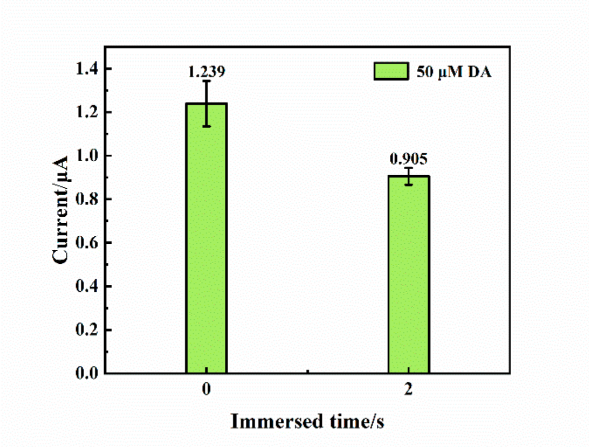

Figure S6. Current response of the 3D rGO-Ti_3C_2 electrodes before and after immersing in 40 mg mL^{-1} BSA for DA.

7. Animals

As shown in Figure S7, rat striatum was implanted with electrodes.

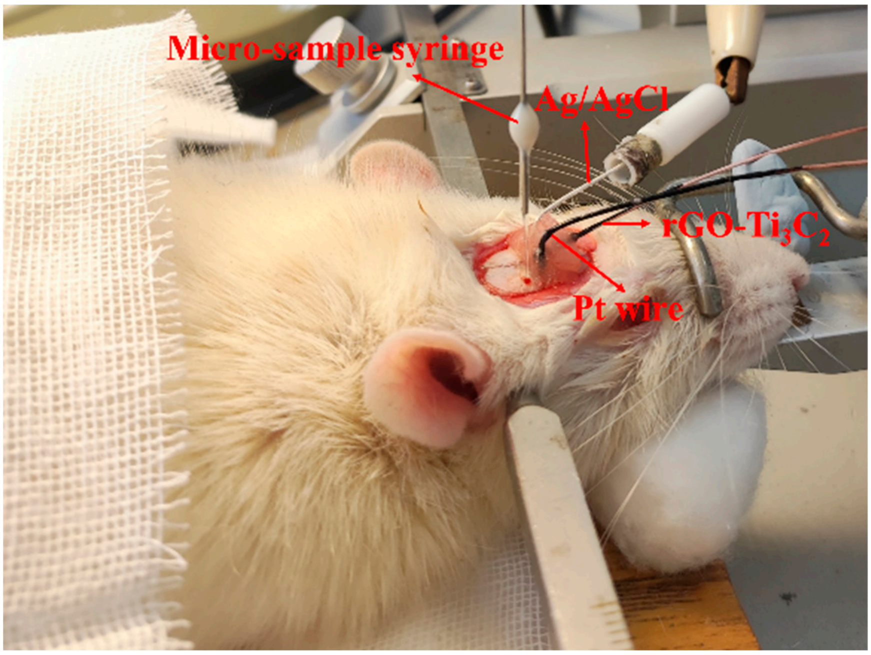

Figure S7. 3D rGO-Ti_3C_2 electrode, Pt wire, Ag/AgCl, and micro-sample syringe were implanted into rat striatum

Reference

1. Chen, S.; Shi, M.; Xu, Q.; Xu, J.; Duan, X.; Gao, Y.; Lu, L.; Gao, F.; Wang, X.; Yu, Y., Ti3C2Tx MXene/nitrogen-doped reduced graphene oxide composite: a high-performance electrochemical sensing platform for adrenaline detection. *Nanotechnology* **2021,** 32, (26).
2. Zhang, L.; Or, S. W., Self-assembled three-dimensional macroscopic graphene/MXene-based hydrogel as electrode for supercapacitor. *APL Materials* **2020,** 8, (9).
3. Sun, Y.; Wang, B.; Deng, Y.; Cheng, H.; Li, X.; Yan, L.; Li, G.; Sun, W., Reduced graphene oxide/titanium carbide MXene nanocomposite-modified electrode for electrochemical hemoglobin biosensor. *Journal of the Chinese Chemical Society* **2021,** 68, (12), 2326-2336.
